# Ovine Mesenchymal Stem Cell Chondrogenesis on a Novel 3D-Printed Hybrid Scaffold In Vitro

**DOI:** 10.3390/bioengineering11020112

**Published:** 2024-01-24

**Authors:** Arianna De Mori, Agathe Heyraud, Francesca Tallia, Gordon Blunn, Julian R. Jones, Tosca Roncada, Justin Cobb, Talal Al-Jabri

**Affiliations:** 1 School of Pharmacy and Biomedical Science, University of Portsmouth, St Micheal’s Building, White Swan Road, Portsmouth PO1 2DT, UK; gordon.blunn@port.ac.uk; 2 Department of Materials, Imperial College London, South Kensington Campus, London SW7 2AZ, UK; 3 Trinity Center for Biomedical Engineering, Trinity Biomedical Science Institute, Trinity College Dublin, 152-160 Pearse Street, DO2 R590 Dublin, Ireland; roncadat@tcd.ie; 4 Department of Surgery and Cancer, Imperial College London, London SW7 2AZ, UK

**Keywords:** sheep, mesenchymal stem cells, chondrogenesis, polycaprolactone, 3D printing, silica, tetrahydrofuran, human, cell differentiation

## Abstract

This study evaluated the use of silica/poly(tetrahydrofuran)/poly(ε-caprolactone) (SiO_2_/PTHF/PCL-diCOOH) 3D-printed scaffolds, with channel sizes of either 200 (SC-200) or 500 (SC-500) µm, as biomaterials to support the chondrogenesis of sheep bone marrow stem cells (oBMSC), under in vitro conditions. The objective was to validate the potential use of SiO_2_/PTHF/PCL-diCOOH for prospective in vivo ovine studies. The behaviour of oBMSC, with and without the use of exogenous growth factors, on SiO_2_/PTHF/PCL-diCOOH scaffolds was investigated by analysing cell attachment, viability, proliferation, morphology, expression of chondrogenic genes (RT-qPCR), deposition of aggrecan, collagen II, and collagen I (immunohistochemistry), and quantification of sulphated glycosaminoglycans (GAGs). The results showed that all the scaffolds supported cell attachment and proliferation with upregulation of chondrogenic markers and the deposition of a cartilage extracellular matrix (collagen II and aggrecan). Notably, SC-200 showed superior performance in terms of cartilage gene expression. These findings demonstrated that SiO_2_/PTHF/PCL-diCOOH with 200 µm pore size are optimal for promoting chondrogenic differentiation of oBMSC, even without the use of growth factors.

## 1. Introduction

Articular cartilage, a specialised connective tissue crucial for joint function and mobility [1] is a viscoelastic tissue composed of chondrocytes embedded within a complex extracellular matrix of collagens, proteoglycans, and glycoproteins [2]. Its intricate architecture and limited self-healing capacity (due to its avascularity and low cell turnover) make it susceptible to chondral lesions [3,4]. These localised areas of damage can occur due to acute or repetitive trauma, age-related changes, genetic disorders, or other joint-related diseases. Although the true incidence of cartilage lesions is unknown, studies report articular defects in 60–66% of knees undergoing arthroscopy [5]. Left untreated, chondral lesions can lead to chronic pain, joint disability, inflammation, and early-onset osteoarthritis, significantly impacting quality of life [6,7,8,9].

The current treatment for defects smaller than 2 cm^2^ involves microfracture, releasing bone-marrow-derived mesenchymal stem cells (MSC) through subchondral drilling [10,11]. However, the regenerated cartilage tends to be fibrocartilage [2,12] with inferior mechanical properties. For larger defects, autologous chondrocyte implantation (ACI) and matrix-induced chondrocyte implantation (MACI) use a two-step process with harvested chondrocytes from the patient. In particular, ACI involves implantation under a periosteal flap, while MACI uses a matrix without a flap [13,14,15,16,17,18]. Autologous matrix-induced chondrogenesis (AMIC) is a one-step alternative treatment combining subchondral microfracture (which releases MSC) with a collagen I/III matrix for covering the blood clot and its MSC [19]. Despite an encouraging number of positive clinical short-to-medium-term outcomes, the long-term success remains debatable [20,21]. For instance, Mancini and Fontana found that 5 years after surgery, in 57 patients (26 of whom received MACI and 31 received AMIC), no difference was reported between AMIC and MACI treatments in acetabular chondral lesions [22]. Overall, the low durability of the regenerated cartilage, often fibrocartilage, is one of the biggest challenges for ACI, MACI, and AMIC [2]. As a result, there is extensive ongoing research into novel strategies, including the fabrication of three-dimensional (3D) scaffolds for enhancing the regeneration of cartilage in vivo [12,22,23].

Three-dimensional printing technology enables the production of resorbable products with designed morphology, fostering an environment crucial for maintaining the chondrocyte phenotype [24]. Biomechanical signals and scaffold architecture play a vital role in chondrogenic differentiation, with studies revealing that smaller scaffold pore size (20–150 µm) enhances chondrogenesis compared to larger pores (150–500 µm) in (collagen and polyurethane) scaffolds [20,25,26,27,28,29]. This improvement is believed to stem from improved intracellular signalling in the smaller pores. However, other studies, using gelatin scaffolds, have demonstrated conflicting results, suggesting that larger pore sizes (250–500 µm) can enhance chondrocyte proliferation and extracellular matrix secretion [25,26]. Similarly, conflicting findings arise from studies involving poly(ε-caprolactone) (PCL) scaffolds, with pore size in the range 88–405 µm, suggesting that larger pores favour chondrocyte adhesion and growth [27]. The effect of scaffold pore size on cell behaviour may be attributed to specific cell–scaffold interactions such as surface chemistry, space, stiffness, and scaffold degradation. Considering what has been said above, drawing conclusions on the ideal pore size for chondrogenesis becomes challenging due to the heterogeneity among these studies, which encompass different materials, designs, and cell types [28,29,30].

Recently, a novel class of medical devices, that is, inorganic/organic hybrid biomaterials like silica/poly(tetrahydrofuran)/poly(ε-caprolactone), has been developed to mimic the natural stiffness and friction coefficient of cartilage. Silica, chosen for its biodegradability and ability to form covalent bonds with the organic networks, has been shown to enhance compressive yield strength in silica/poly(tetrahydrofuran)/poly(ε-caprolactone). While natural articular cartilage possesses a compressive strength of 22–37 MPa, Tallia et al. showed that the silica/poly(tetrahydrofuran)/poly(ε-caprolactone) scaffolds with 250 µm pore channels have a compressive yield strength of 1.2 ± 0.2 MPa [31]. Considering the involvement of human mesenchymal cells (hMSC) in microfracture and AMIC surgical techniques, Li et al. also investigated the impact of pore size in 3D-printed SiO_2_/PTHF/PCL-diCOOH on hMSC chondrogenic differentiation. Optimal results were observed with ~250 µm channels, fostering dense cell populations and increased production of collagen type II, aggrecan, and GAGs compared to 100 µm and 500 µm pores. Larger 500 µm pores indicated de-differentiation to fibroblasts, probably due to reduced cell–cell interactions [32].

While results for human MSC culture on the hybrid scaffolds are promising, evaluating the chondrogenic response in vivo on SiO_2_/PTHF/PCL-diCOOH scaffolds is crucial. Although sheep are considered a suitable large animal model for osteochondral defects, previous in vitro studies comparing human and ovine stem cells showed that the latter exhibit different properties from their human counterpart. For instance, Haddouti et al. conducted a comparative study of mesenchymal stem cells derived from corresponding sources in humans and sheep (adipose tissue, femoral marrow fat, and bone marrow). MSC from both species revealed robust growth behaviour and strong immunomodulatory capacity. In addition, common positive and negative surface markers were identified for humans and sheep. However, osteogenic differentiation was slower in oMSC compared to hBMSC [33]. In contrast, Westerkowsky et al. observed that ovine bone marrow stromal cells (oBMSC) exhibited a stronger osteogenic potential and possessed a significantly higher doubling time than human bone marrow stromal cells (hBMSC). However, chondrogenic differentiation was less efficient in oBMSC than in hBMSC [34]. Overall, these studies show that a systematic approach and comparative studies are necessary to interpret the in vivo response in the different species.

The aim of this study is to determine if ovine mesenchymal stem cells behave similarly to what was previously observed for hBMSC when cultured on 3D-printed SiO_2_/PTHF/PCL-diCOOH scaffolds. In this paper, we investigated the influence of 3D-printed scaffolds, with 200 µm (SC-200) and 500 µm pores (SC-500), on the chondrogenic differentiation of primary oBMSC in basal (BM) or chondrogenic medium (CM). The hypothesis is that oBMSC will behave on SiO_2_/PTHF/PCL-diCOOH with pore sizes of 200 and 500 µm in a similar manner to the observations in our previous publication, providing validation for the use of SiO_2_/PTHF/PCL-diCOOH for prospective in vivo ovine studies.

## 2. Materials and Methods

### 2.1. Hybrid Synthesis

Silica/poly(tetrahydrofuran)/poly(ε-caprolactone) (SiO_2_/PTHF/PCL-diCOOH) sol–gel hybrids were prepared as previously described [31]. The first step was to synthesise dicarboxylic acid (PCL-diCOOH) from PCL diol (M_n_ = 530 Da) via the 2,2,6,6-Tetramethyl-1-piperidinyloxy (TEMPO) oxidation process. PCL-diCOOH (1 mol), (3-glycidyloxypropyl) trimethoxysilane (GPTMS, 2 mol), and boron trifluoride diethyl etherate (BF_3_·OEt_2_, 0.5 mol) were then mixed in THF (100 mL mg^−1^ wrt PCL-diCOOH) (Merck Life Science, Gillingham, UK). This organic precursor solution was mixed for 1.5 h at room temperature, allowing polymerisation of THF to occur, forming PTHF. In parallel, the hydrolysis of tetraethyl orthosilicate (TEOS, 80 wt.% w.r.t. PCL-diCOOH) (Merck Life Science, Gillingham, UK) was performed using stoichiometric volume of deionised water in acidic condition with hydrochloric acid (1 M HCl, 1/3 *v*/*v* wt deionised water) (Merck Life Science, Gillingham, UK). Once hydrolysed, the inorganic silica precursor solution was added dropwise to the organic solution and stirred at room temperature for a further 30 min in a sealed perfluoroalkoxy alkane container. This formed the hybrid sol. Further THF evaporation was achieved by opening the lid under continuous stirring to accelerate the gelation process and achieve appropriate viscosity. This hybrid ink was then transferred into 3 mL Luer-lock syringes for 3D printing hybrid scaffold via direct ink writing. The hybrid ink was stored at −82 °C.

### 2.2. Direct Ink Writing for Hybrid Scaffold Fabrication and Characterisation

This hybrid ink was 3D printed using direct ink writing, utilising the sol–gel gelation process as previously described [31]. The ink was thawed at room temperature (10–15 min) and loaded into a direct ink writing machine (Robocaster, 3d Inks LLC, Tulsa, OK, USA) with a tapered tip (Nordson EFD, Westlake, OH, USA). The software Robocad (3d Inks LLC, Tulsa, OK, USA) was used to control the printing parameters to create a porous scaffold. Utilising the continuous gelation process of the sol–gel hybrid ink, a 1 h window was achieved once the correct printing viscosity was reached, allowing for smooth extrusion and no collapsing of the struts once printed. A tapered nozzle with an internal diameter of 0.20 mm, a strut spacing of 0.60 mm, z-spacing of 0.21 mm, and printing speed of 10 mm s^−1^ were used. The scaffolds were printed with side dimensions of 10.8 mm and 10 layers. These were then aged and dried in a 40 °C oven for 3 and 7 days, respectively. Once dried, the samples were cut to 5 mm-diameter scaffold discs (height: 1–1.4 mm) using biopsy punches (Kai Medical, Solingen, Japan) and washed in deionised water three times for 10 s to remove by-products. Scaffolds were transferred to glass vials (Supelco, Inc., Bellafonte, PA, USA) and sterilised with 50 kGy γ-irradiation. The architecture of the scaffolds was investigated using scanning electron microscopy (SEM JEOL 6010LA) after gold sputter coating, as previously described [32].

### 2.3. Isolation and Characterisation of Ovine Mesenchymal Stem Cells

Ovine MSC were obtained from adult (3 years old) female sheep weighing 70 kg. A bone marrow aspirate was taken from the iliac crest. All procedures were carried out in accordance with the UK’s Animals (Scientific procedures) Act, 1986, at the Royal Veterinary College, North Mymms. The project was approved by the animal welfare and ethical review body of the Royal Veterinary College, and the procedures were carried out under a Home Office Project (project licence number P16F4AA0A). Home Office personal licences were held by all those taking part in any surgical procedure. Briefly, the aspirate was gently dispensed into a centrifuge tube to obtain an upper layer of bone marrow and a bottom layer of Ficoll. Subsequently, the samples were centrifuged at 300 g for 25 min. The buffy layer was washed in sterile PBS and centrifuged at 300 g for 5 min. The pellet was resuspended in DMEM supplemented with 10% foetal bovine serum (FBS) (Fisher, Loughborough, UK) and penicillin/streptomycin (1%) (Fisher, Loughborough, UK) and plated into a 25 cm^2^ flask. The media was changed after 12 h. Cells were cultured in a humidified atmosphere at 37 °C, 5% CO_2_, and at 80% confluence.

The oBMSC were previously characterised for their phenotype, showing that they were positive for CD29 and CD44, negative for CD45 and CD34, and able to differentiate into adipocytes, osteocytes, and chondrocytes [35]. Cells were frozen in liquid nitrogen and once thawed and cultivated, used at passage 4.

### 2.4. In Vitro Cell Culture

Cells were grown, under aseptic conditions, in basal medium (BM), consisting of DMEM (high glucose, GlutaMAX, pyruvate manufacturer) (Fisher, Loughborough, UK) supplemented with 10% heat-inactivated foetal bovine serum and 1% penicillin–streptomycin. The cells were cultured at 37 °C in a humidified incubator under 5% CO_2_. Culture media was changed 2–3 times per week. At 80–90% confluence, the media was removed, and the cells washed with Dulbecco’s phosphate-buffered saline (without calcium and magnesium) (Fisher, Loughborough, UK). The cells were detached following the incubation with 0.25% trypsin-EDTA (Fisher, Loughborough, UK).

### 2.5. Cell Seeding on Scaffolds

Hybrid scaffold discs (5 × 2.5 mm) were initially pre-washed in an ascending ethanol series (50%, 70%, and 100%) for 5 min each (Fisher, Loughborough, UK). Then, they were rinsed with sterile deionised water (5 min) and coated with sterile heat inactivated FBS (4 h, R.T.), under gentle agitation. The FBS coating was used to improve cell attachment. Excess FBS was then removed, and the scaffolds rinsed once with deionised water [36,37,38]. Finally, the constructs were air-dried under laminar flow overnight. Seeding was carried out the following day. Changes in surface wettability of the materials was assessed by contact angle, as previously reported [39].

Ovine bone marrow stem cells were seeded on the scaffolds using the following method: initially, a suspension of 25,000 cells/µL was prepared, and a total of 10,000,000 cells were seeded on each scaffold. Half of the cells in 20 µL were seeded on one side of the scaffold and then incubated for 90 min (5% CO_2_ and 37 °C). The same procedure was repeated for the opposite side of the constructs. Then, the scaffolds were gently covered with the complete medium and incubated (5% CO_2_ and 37 °C). After 24 h, the scaffolds were transferred (with autoclaved sterile forceps) to a 48-well plate (low cell attachment plates) (Sarstedt, Loughborough, UK) and supplemented with 800 µL of either basal (BM) or chondrogenic medium (CM) (StemPro™ Chondrogenesis Differentiation Kit) (Fisher, Loughborough, UK). Cells in 6-well plates (2D), on either the tissue culture plastic surface or on plastic coverslips, were used as controls.

### 2.6. Cellular Attachment and Viability

Cells seeded on scaffolds were allowed to adhere for 24 h on both the scaffolds and plates. Subsequently, samples were rinsed once with sterile PBS. Cell metabolic activity was assessed using a resazurin assay, where an increase in the production of the redox product, detected through a resorufin colour change, indicates a higher metabolic activity and, potentially, a higher cell number of viable cells [40]. Briefly, a filter-sterile 0.1 mM resazurin salt solution (Merck Life Science, Gillingham, UK) in basal medium (working solution) was added to each well. The plates were then incubated (5% CO_2_ and 37 °C) for 2 h. The working solution, incubated in the same conditions, served as a blank. Then, 100 µL aliquots were transferred to a new 96-well plate and the fluorescence was immediately read using a plate reader (ex/em 540/585 nm) (SpectraMax i3x) (Molecular Devices, San Jose, CA, USA). The following formula was used to determine the percentage of cells attached to the scaffolds:

$$\left(\frac{F_{sc}}{F_{con}}\right)\times 100,$$

where F_sc_ is the fluorescence read in the resazurin solution in contact with scaffolds (minus the blank) and cells, and F_con_ is the one of the cells grown in 2D as control (minus the blank).

The resazurin assay was carried out as described above on days 7, 14, and 21 after treatment.

### 2.7. Live/Dead Staining

The viability of oBMSC on scaffolds and coverslips was analysed on days 7 and 21 using double staining with ethidium homodimer-1 and calcein-AM in PBS (4 and 2 µM, respectively) (Merck Life Science, Gillingham, UK). Briefly, the scaffolds and coverslips were moved to a new plate and washed, once, with PBS. Subsequently, 500 µL of working solution was added to each well, and the plates were incubated for 1 h (5% CO_2_ and 37 °C). Afterward, the samples were gently rinsed twice with PBS and imaged with a confocal laser microscope (LSM 710, Zeiss, Oberkochen, Germany) at 488 and 543 nm. Cell viability was quantified using ImageJ software (National Instruments, Austin, TX, USA). Cells were manually counted using the multi-point tool. The percentage of viable cells were calculated according to the following equation:

$$\left(\frac{Live\;cells}{Total\;number\;of\;cells}\right)\times 100.$$

### 2.8. Cellular Morphology by Scanning Electron Microscopy

On days 7 and 21, samples were rinsed with 0.1 M cacodylate buffer and fixed with 2.5% glutaraldehyde in 0.1 M cacodylate (Merck Life Science, Gillingham, UK) overnight at 4 °C. Following fixation, the samples were washed 3 times with deionised water and dehydrated in an ethanol series (20 min each at 50%, 70%, 90%, and 100%) followed by treatment with 1:2 and 2:1 HMDS (hexamethyldisilane): EtOH (20 min) and 100% HMDS (overnight) (Merck Life Science, Gillingham, UK). The samples were then mounted on stubs and gold- sputtered coated using a Polaron e500 (Quorum Technologies, Lewes, UK). Imaging was performed with an SEM with an accelerating voltage of 20 kV (EVO ma10, Zeiss, White Plains, NY, USA).

### 2.9. RNA Extraction and RT-qPCR

Qiazol lysis reagent (Qiagen, Manchester, UK) was used to lyse cells on days 7 and 21. Cell content was homogenised using a sterile 25G needle and centrifuged at 18,000× *g* for 2 min at room temperature. The supernatant was processed using a RNeasy PLUS micro kit for RNA isolation (Qiagen, Manchester, UK). RNA was quantified using a nanodrop (Nanodrop ND-1000) and stored at −80 °C until use. Complementary DNA (cDNA) was obtained from RNA (250–500 ng from each sample) using a High-Capacity cDNA Reverse Transcription Kit (Fisher, Loughborough, UK) on a thermal cycler (T100, Bio Rad, Watford, UK), according to the manufacturer’s instructions. RT-qPCR was performed in a LightCycler96 (Roche, Burgess Hill, UK) using SSO Universal SYBR Green Supermix (Fisher, Loughborough, UK), and data were analysed using the LightCycler SW 1.1 analysis software (Roche, Penzberg, Germany). Relative gene expression was calculated using the comparative 2-ΔΔCt (expressed as a fold change). GAPDH was used as a housekeeping gene. The used primers were: Glyceraldehyde 3-phosphate dehydrogenase (*GAPDH*), Runt-related transcription factor 2 (*RUNX2)*, SRY-Box Transcription Factor 9 (*SOX9*), SRY-Box Transcription Factor 5 (*SOX5*), collagen type 1, alpha 1 (*COL1A1*), collagen type 2, alpha 1, *COL2A1*, and Aggrecan (*ACAN*) (Eurofins Genomics, Ebersberg, Germany). The primer sequences have been reported in a previous publication [35].

### 2.10. Immunohistochemistry

At 21 days of culture, the specimens underwent immunohistochemical analysis to assess the expression of collagen I, collagen II, and aggrecan. Samples were fixed in 4% paraformaldehyde (PFA) for 20 min. The scaffolds were not sectioned before staining due to the temperature sensitivity of PCL (both to heat and freezing) and its solubility in organic solvents. Following two washes in PBS (1×), samples were permeabilised with 0.2% Triton X-100 (Merck Life Science, Gillingham, UK) in PBS for 10 min. Non-specific binding was blocked with bovine serum albumin (BSA) (2% in PBS, 1 h, R.T.) (Merck Life Science, Gillingham, UK). Next, primary antibodies were added: anti-collagen I antibody (1:400) (ab138492, Abcam), anti-collagen II antibody (1:300) (ab138492), and anti-aggrecan (1:300) (ab3778, Abcam) (Abcam, Cambridge, UK). The samples were incubated at 4 °C overnight. The samples were then washed with PBS (three times) and the secondary antibodies were added: Goat Anti-Rabbit IgG H&L (Alexa Fluor^®^ 594) (ab150080) and sheep Anti-Mouse IgG (whole molecule) F(ab’)2 fragment-FITC (1:300) (Abcam, Cambridge, UK) in BSA 1%, for 1 h at room temperature. After rinsing with PBS (three times), the samples were stained with 4′,6-diamidino-2-phenylindole (DAPI) (5 µg/mL) (Merck Life Science, Gillingham, UK) in PBS for nuclear localization, for 10 min. The samples were rinsed again (twice) with deionized water. Images were taken using confocal fluorescence microscopy (ZEISS LSM 710, ZEISS Research Microscopy Solutions) at 405, 488, and 594 nm.

### 2.11. Sulphated Glycosaminoglycan (sGAGs) Quantification

On days 7 and 21, the samples were fixed in PFA 4% overnight. The samples were then incubated in 3% acetic acid (Fisher, Loughborough, UK) for 3 min. Alcian blue solution (1%, in 3% acetic acid) (Fisher, Loughborough, UK) was added to the samples for 60 min, and excess solution was removed by rinsing with deionised water. Subsequently, guanidine hydrochloride solution (8 M) (Merck Life Science, Gillingham, UK) was added, and the samples were incubated overnight at 4 °C. An aliquot from each well was transferred to a 96-well plate. The absorbance at 600 nm was measured using a spectrophotometer (SpectraMax i3x, Molecular Devices) to quantify the sGAGs content.

For DNA content quantification, nuclei were stained with Harris Haematoxylin (Merck Life Science, Gillingham, UK) for 5 min, and excess staining was removed with 2 washes in deionised water. Subsequently, a mixture of 100% ethanol and 99% acetic acid (75:25) (Fisher, Loughborough, UK) was added, and the samples were incubated at 45 °C (4 h). Aliquots from each sample were transferred to a 96-well plate. The absorbance was read at 450 nm using a spectrophotometer to quantify DNA content.

#### Statistical Analysis

All experiments were conducted using isolates from 3 different sheep, and each treatment was the result of three replicate cultures for each animal (*n* = 3). Data are expressed as the mean ± standard deviation (SD). Normality was initially checked with a Shapiro–Wilk normality test (level of significance = 0.05). Comparison between groups was assessed as indicated in the caption of each figure. The statistical analyses were performed with GraphPad Prism version 10.0.0 (GraphPad Software, Boston, Boston, MA, USA, www.graphpad.com) (accessed on 10 December 2023). Data were considered significant when *p* < 0.05.

## 3. Results

### 3.1. Contact Angle and Cell Attachment

**Figure 1:**
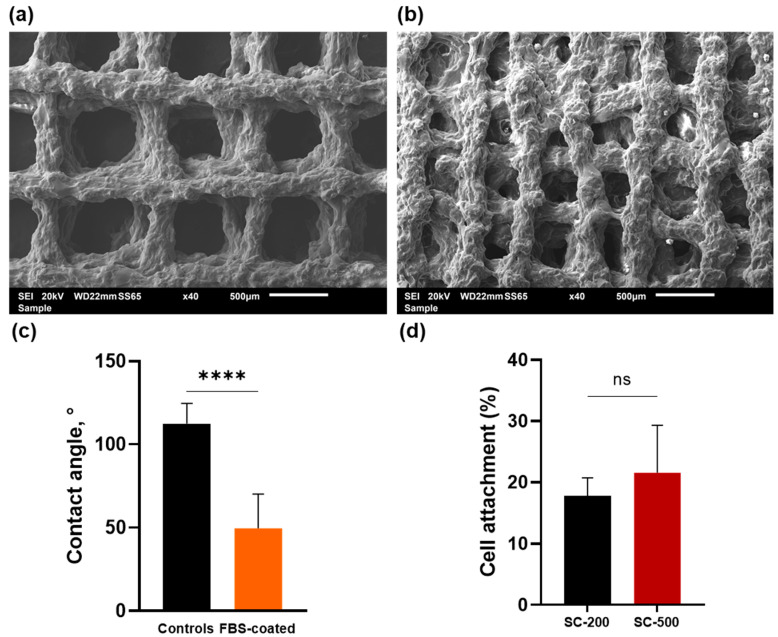
SEM images of 3D-printed SiO_2_/PTHF/PCL-diCOOH scaffolds with pore size: (**a**) 500 µm; (**b**) 200 µm. (**c**) Water contact angle measurements for SC-200 scaffolds before and after FBS coating. Statistical analysis was performed with an unpaired *t*-test (****, *p* < 0.0001). (**d**) Cell attachment on SC-200 and SC-500 scaffolds, determined by resazurin assay, on day 1 after seeding. Error bars denote standard deviation (n = 3). Statistical analysis was performed with an unpaired *t*-test (ns= not significant, where *p* ≥ 0.05).

In this study, scaffolds with two different pore sizes were tested: 500 (SC500) and 200 (SC200) µm (Figure 1a,b, respectively). Specifically, the average channel width was 512.3 ± 99.4 μm for SC-500 and 241.4 ± 61.5 μm for SC-200. Additionally, despite maintaining the 3D grid-like lattice architecture, the 3D printing directly from sol–gel produced struts with irregular surface. To enhance cell adhesion to the scaffolds, the materials were pre-treated with FBS. Thereafter, the hydrophilicity of the surface, before and after coating with FBS, was evaluated via contact angle measurements on SC-200. The hydrophilicity of the scaffolds was enhanced significantly after surface treatment with FBS (from an average Theta degree of 112.41 to 49.51) (*p* < 0.0001) (Figure 1c).

Based on the resazurin fluorescence reading, the percentage of cell adhesion on day 1 after seeding was determined to be 17.82 ± 2.35% for SC-200 and 21.55 ± 6.33% for SC-500, with no statistical difference observed (*p* > 0.05) (Figure 1d).

### 3.2. Cell Metabolic Activity and Viability

**Figure 2:**
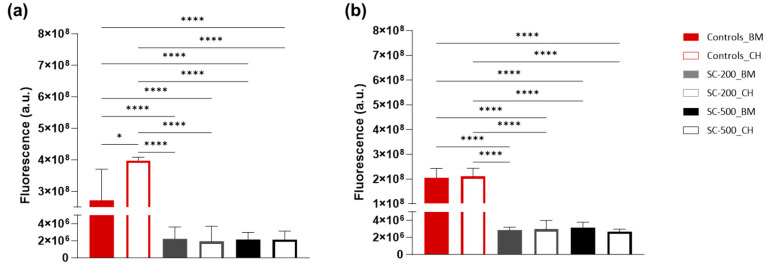
The cell metabolic activity of oBMSC in 2D controls and on SiO_2_/PTHF/PCL-diCOOH scaffolds (pores of 200 μm, SC-200; and pores of 500 μm, SC-500) on days 7 (**a**) and 21 (**b**) after seeding, as determined by resazurin assay. Cells were grown with either basal medium (BM) or chondrogenic medium (CH). Error bars denote standard deviation (n = 3). One-way ANOVA was used to compare the different groups at each time point (7 and 21 days): * *p* < 0.05 and, **** *p* < 0.0001.

On both days 7 and 21, the 2D controls exhibited significantly higher metabolic activity than cells grown on scaffolds (one-way ANOVA, *p* < 0.0001)(Figure 2a,b) For the cells grown in 3D, the addition of chondrogenic medium did not significantly alter the fluorescence emission. No difference in the resorufin production was found between the two different pore sizes. Finally, the only statistical difference between days 7 and 21 was recorded for cells grown in 2D with chondrogenic medium (two-way ANOVA, *p* < 0.0001). The results suggest the scaffolds were not toxic to the cells.

**Figure 3:**
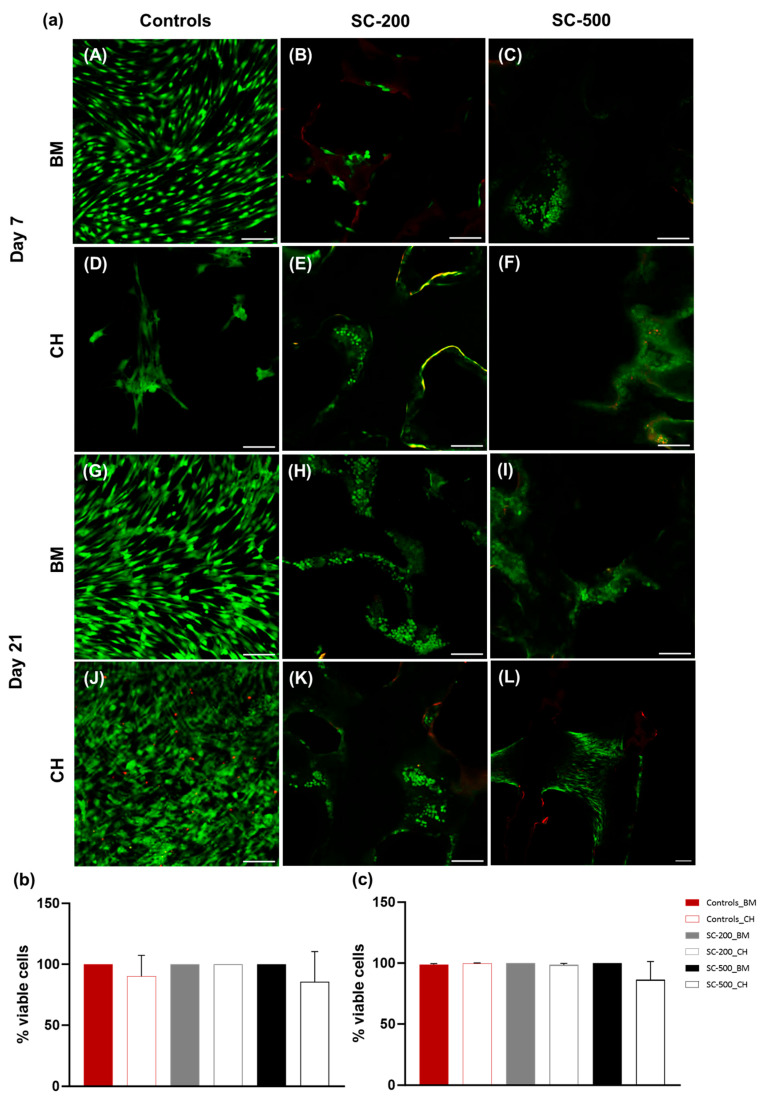
(**a**) Representative images of live/dead staining of oBMSC, grown in either basal (BM) or chondrogenic medium (CH), on days 7 and 21. Cells were imaged on either coverslip (**A**,**D**,**G**,**J**), SC-200 (**B**,**E**,**H**,**K**) or SC-500 (**C**,**F**,**I**,**L**) by a confocal microscope. Scale bar is 80 µm. Quantitative analysis of live and dead cells on coverslips and controls on days 7 (**b**) and 21 (**c**). Results are presented as a mean ± SD (n = 3). Error bars denote standard deviation. One-way ANOVA was used to compare the different groups at each time point (7 and 21 days): *p* > 0.05.

Confirmation of the non-toxic nature of the scaffolds was obtained through a live and dead assay, revealing that over 85% of the cells remained viable across all the treatment conditions and time points (Figure 3a–c). On both days 7 and 21, no statistical difference was determined among different groups (one-way ANOVA, *p* < 0.0001). No difference among the different time points (two-way ANOVA, *p* > 0.05).

### 3.3. Cell Morphology

**Figure 4:**
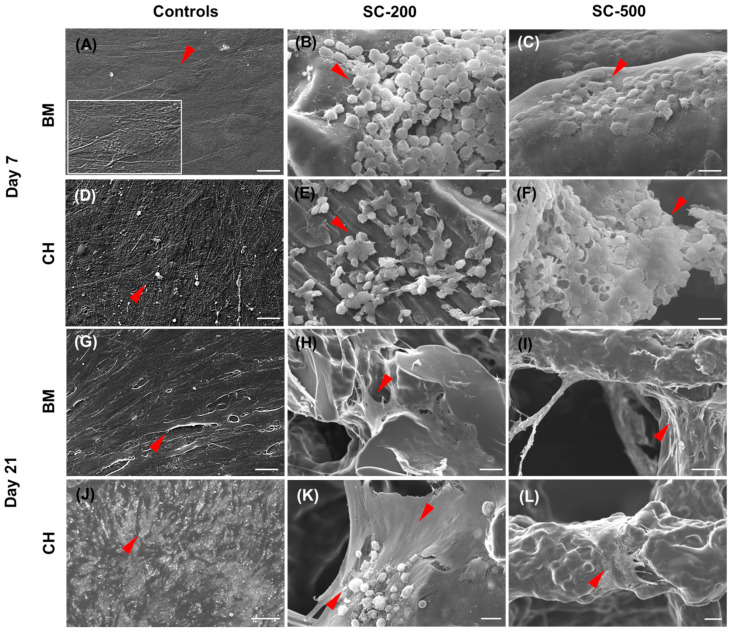
SEM images representing oBMSC cultured on coverslips (**A**,**D**,**G**,**J**) and scaffolds (**B**,**C**,**E**,**F**,**H**,**I**,**K**,**L**), at days 7 and 21. Ovine bone marrow stem cells were grown with either basal medium (BM) or chondrogenic medium (CH). Red arrows indicate cells on coverslips and scaffolds. (**A**) includes a zoomed-in photo (white picture border) of the stem cell indicated by the red arrow. All the images have a scale bar of 20 µm, except for SC-200/BM/Day 21 (40 µm), SC-500/BM/Day 21 (100 µm), and SC-500/CH/Day 21 (50 µm).

The morphology, adhesion, and distribution of oBMSC on coverslips and scaffolds was assessed using scanning electron microscopy (Figure 4). SEM images revealed that the cells adhered to both the 3D scaffolds (on/near the struts) and the coverslips. Interestingly, when grown on 3D scaffolds without FBS coating, the oBMSC presented a spherical shape within the initial 24 h (Figure S1). In contrast, when FBS was used for pre-coating the samples, the cells showed both rounded and fibroblast-like shapes. By day 7, most of the oBMSC grown on coated scaffolds (regardless of the use of chondrogenic medium) presented a rounded shape, a common morphology of chondrocytes (Figure 4). On day 21, most of the oBMSC cultured on SC-200 with basal medium assumed an elongated shape with extended pseudopodia, while some cells cultured in chondrogenic medium were rounded and others were elongated. Concurrently, the 2D controls displayed an elongated shape when grown in basal medium. Interestingly, when cultivated with the chondrogenic medium, these 2D control cells assumed a more rounded shape and accumulated an extracellular matrix which appeared to be enriched with mineral deposits. At the same time point, oBMSC cultured on SC-500 presented a fibroblast-like shape, independent of the treatment [32].

### 3.4. Relative Gene Expression

**Figure 5:**
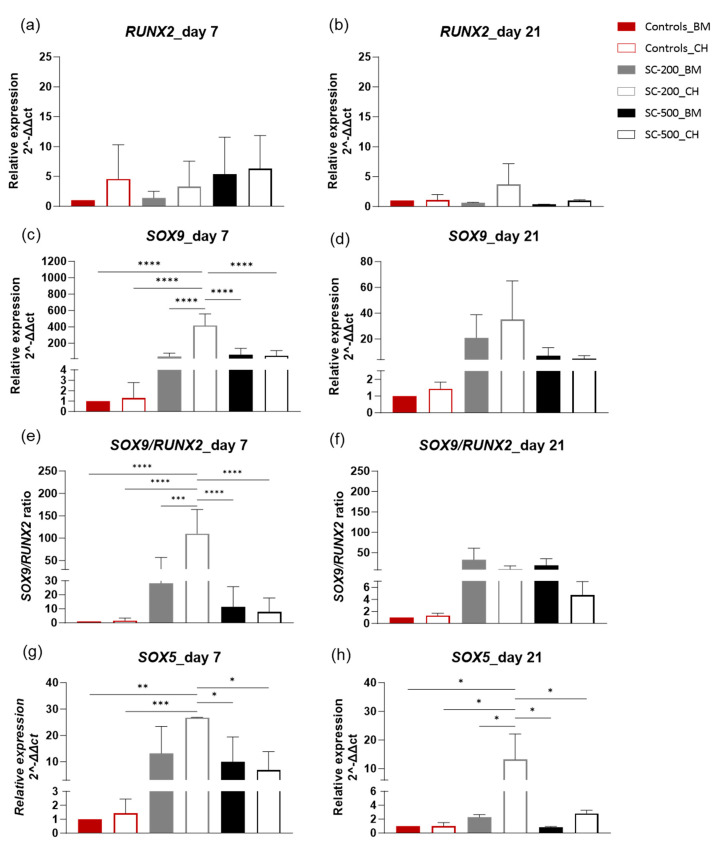
Gene expression of oBMSC on 2D in either BM or CH, and on SC-200 and SC-500 with either BM or CH. Relative gene expression of *RUNX2* on days 7 (**a**) and 21 (**b**); relative gene expression of *SOX9* on days 7 (**c**) and 21 (**d**); *RUNX2*/SOX9 ratios on days 7 (**e**) and 21 (**f**); relative gene expression of *SOX5* on days 7 (**g**) and 21 (**h**). Messenger RNA data are presented as a fold change expression relative to the 2D controls grown with basal medium (BM). Results are presented as a mean ± SD (n = 3). Comparison among groups was assessed by ordinary one-way ANOVA (*p* < 0.05) (* *p* < 0.05, ** *p* < 0.01, *** *p* < 0.001, **** *p* < 0.0001).

RT-qPCR analyses compared the expression of genes involved in both chondrogenesis (*COL2A1*, *SOX5*, *SOX9*, and *ACAN*) and osteogenesis (*RUNX2* and *COL1A1*) [41]. In the experiment, there was an upregulation of *RUNX2* expression (Figure 5a,b) in comparison to the 2D controls in basal medium, for all the scaffold groups, but the difference was not significant on days 7 and 21. SC-500 in chondrogenic medium showed the highest expression of *RUNX2* on day 7 (6-fold), followed by SC-500 in basal medium (5-fold), 2D controls in CH (ca. 5-fold), and SC-200 in BM (>1-fold change). On day 21, the expression of *RUNX2* decreased for SC-200 in BM, SC-500 in BM, SC-500 in CH, and 2D controls in CH (*p* > 0.05), but slightly increased for SC-200 in CH (*p* > 0.05). No statistical difference was seen when comparing day 7 with day 21 (Figure S2a).

Sry-related transcription factor-9 (*SOX9*) was upregulated on day 7 in all the groups (Figure 5c), even without the addition of chondrogenic growth factors. On day 7, the pore size influenced the expression of *SOX9*, particularly on SC-200 in chondrogenic medium, showing over-400-fold increase compared to the 2D controls in basal medium. On the other hand, *SOX9* expression was ca. 49-fold higher on SC-500 in chondrogenic medium. Furthermore, the *SOX9* expression was higher for SC-500 than for SC-200 (61- vs. 39-fold, respectively), in the basal medium, on day 7. For the same time point, the *SOX9* expression was ca. 1 for 2D controls in CH and ca. 49 for SC-500 in CH medium. The *SOX9* expression decreased on day 21 compared to day 7 for all conditions. *SOX9* relative expression was higher than 2D controls in BM for 2D controls in CH (ca. 1-fold), for SC-200 in BM 921-fold), SC-200 in CH (35-fold), for SC-500 in BM (7-fold), and for SC-500 in CH (4-fold) (*p* > 0.05). No statistical difference (*p* > 0.05) was observed in the *SOX9* expression between the two time points for all the studied groups. The only exception was recorded for SC-200 treated with chondrogenic medium that presented a significant difference between day 7 (416-fold increase) and day 21 (35-fold increase) (*p* < 0.001). 

The analysis of the *SOX9* and *RUNX2* ratios (Figure 5c) revealed that all the oBMSC seeded on the scaffolds shifted their gene expression towards the chondrogenic lineage, even without the use of growth factors. On day 7, the *SOX9/RUNX2* ratio was 28 and 11 for SC-200 and SC-500 in basal medium and 110 and 8 in chondrogenic medium, respectively. The *SOX9/RUNX2* ratio was positive for the 2D controls in CH too. Furthermore, SC-200 in chondrogenic medium showed a statistically significant higher shift towards the chondrogenic lineage than the 2D controls in BM (*p* < 0.0001), 2D controls in CH (*p* < 0.0001), SC-500 in BM (*p* < 0.0001), and SC-500 in CH (*p* < 0.00001). Interestingly, SC-200 in BM had a higher *SOX9/RUNX2* ratio than SC-500 in both BM and CH. Chondrogenic gene expression remained predominant, in comparison to the osteogenic route, on day 21, for all the treatments (Figure 5d). In particular, the ratios were 33 and 19 for SC-200 and SC-500 in basal medium, and 9 and 5 in chondrogenic medium, respectively (*p* > 0.05). No statistical difference was recorded between days 7 and 21 (*p* > 0.05) (Figure S2h).

**Figure 6:**
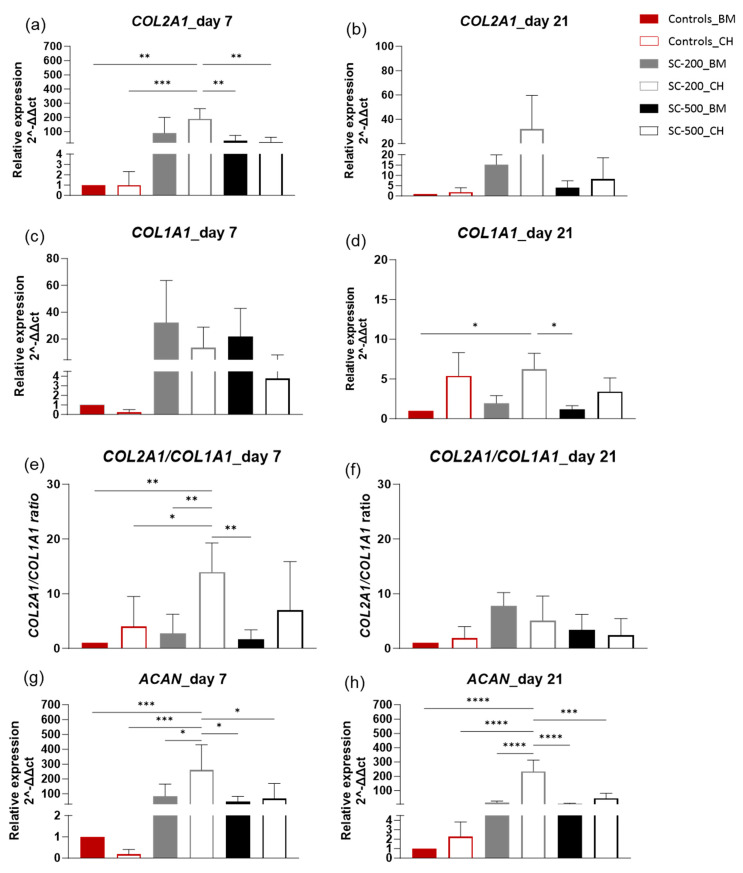
Gene expression of oBMSC on 2D in either BM or CH and on SC-200 and SC-500 with either BM or CH. Relative gene expression of *COL2A1* on days 7 (**a**) and 21 (**b**); relative gene expression of *COL1A1* on days 7 (**c**) and 21 (**d**); *COL2A1/COL1A1* ratios on days 7 (**e**) and 21 (**f**); relative gene expression of *ACAN* on days7 (**g**) and 21 (**h**). Messenger RNA data are presented as fold change expression relative to the 2D controls grown with basal medium (BM). Results are presented as a mean ± SD (n = 3). Comparison among groups was assessed by ordinary one-way ANOVA (*p* < 0.05) (* *p* < 0.05, ** *p* < 0.01, *** *p* < 0.001, **** *p* < 0.0001).

Throughout our study, *SOX5* (Figure 5d) was generally more upregulated on day 7 than on day 21, but the difference was not significant (*p* > 0.05) (Figure S2d). On day 7, the highest levels of gene expression were recorded for SC-200 in CH medium (27-fold), followed by SC-200 in BM (13-fold), SC-500 in BM (10-fold), SC-500 in CH (7-fold), and 2d controls in CH (~1-fold), respectively. SC-200 in CH medium was significantly more expressed than SC-500 in both BM (*p* < 0.05) and CH (*p* < 0.05) and SC-200 in BM (*p* < 0.001). No statistical difference was recorded comparing the other groups. The high expression of the chondrogenic marker, *SOX5*, remained significantly in favour of SC-200 in BM on day 21 (*p* < 0.05). Again, no statistical difference was recorded compared to the other groups on day 21. No statistical difference was determined between days 7 and 21 (Figure S2d). Thereafter, the expressions of ACAN, COL2A1, and COL1A1 was evaluated. The expression of COL2A1 (Figure 6a) significantly increased on day 7 in all the scaffolds, with a greater effect for cells grown on the SC-200 scaffolds in chondrogenic medium, for which the expression increased by 189-fold. The COL2A1 expression on day 7 was significantly higher for SC-200 in CH medium than for 2D controls (p < 0.01), for 2D controls in CH (*p* < 0.001), for SC-500 in BM (*p* < 0.01), and for SC-500 in CH culture fluid (*p* < 0.01). Notably, the *COL2A1* expression was upregulated for both SC-200 and SC-500, even in the absence of chondrogenic growth factors. Again, COL2A1 expression was upregulated for all the groups on day 21 in comparison to the 2D controls in BM, but the difference was not significant (*p* > 0.05) (Figure 6b). No statistical difference was determined between days 7 and 21, except for SC-200 in BM (*p* < 0.05) (Figure S2f).

The expression of *COL1A1* increased in cells on all the scaffolds on day 7 compared to the 2D controls in BM, with SC-200 in basal medium being the one with the highest fold change in *COL1A1* expression (32-fold) (Figure 6c). However, no statistical difference was recorded among groups (*p* > 0.05). The expression of *COL1A1* decreased in all the groups on day 21 (Figure 6d) but remained upregulated compared to the cells grown in 2D cultures of BM, from 32 to 2-fold for SC-200 in BM, from 13- to 5-fold for SC-200 in CH, from 22 to 1-fold for SC-500 in BM, and from 4 to 3-fold for SC-500 in CH. The only exception was recorded for 2D controls in CH, where the *COL1A1* relative gene expression was downregulated on day 7 and was 5-fold more expressed on day 21. No statistical difference was seen when comparing day 7 with day 21 (Figure S2b).

*COL2A1/COL1A1* ratios (Figure 6e,f) were always positive for cells on scaffolds, treatments, and time points, with the highest ratios recorded for SC-200 in chondrogenic medium on day 7 (14-fold). The ratio was significantly different for SC-200 in chondrogenic medium compared to 2D controls in BM (*p* < 0.01) and CH (*p* < 0.05), SC-200 in BM (*p* < 0.01), and SC-500 in BM (*p* < 0.05). No statistical difference was recorded for the different groups at different time points, except for SC-200 CH (*p* < 0.0001) (Figure S2g). The *COL2A1/COL1A1* positive ratios confirmed that the chondrogenic shifting remained predominant, in comparison to the osteogenic route, on day 21 for all the treatments.

The pore size appeared to influence the expression of *ACAN* on day 7 (Figure 6g); in chondrogenic medium, 200 µm pore size promoted a significantly higher expression of aggrecan (260-fold) in comparison to the 2D controls in BM (*p* < 0.001) and CH (downregulated) (*p* < 0.0001) and SC-500 in BM (*p* < 0.01) and in CH (*p* < 0.01). Interestingly, on day 7, ACAN expression was upregulated on both SC-200 (85-fold) and SC-500 (47-fold), even without the addition of chondrogenic growth factors (*p* > 0.05). *ACAN* expression decreased between days 7 and 21 for cells on all the scaffold, but the difference was not significant (*p* > 0.05) (Figure S2f). Finally, no statistical difference was calculated between the different groups on day 21 (*p* > 0.05) (Figure 6h).

Overall, the results suggest that oBMSC can undergo spontaneous chondrogenesis on SC-200 and SC-500 scaffolds, even without the use of exogenous chondrogenic factors.

### 3.5. Immunohistochemistry

**Figure 7:**
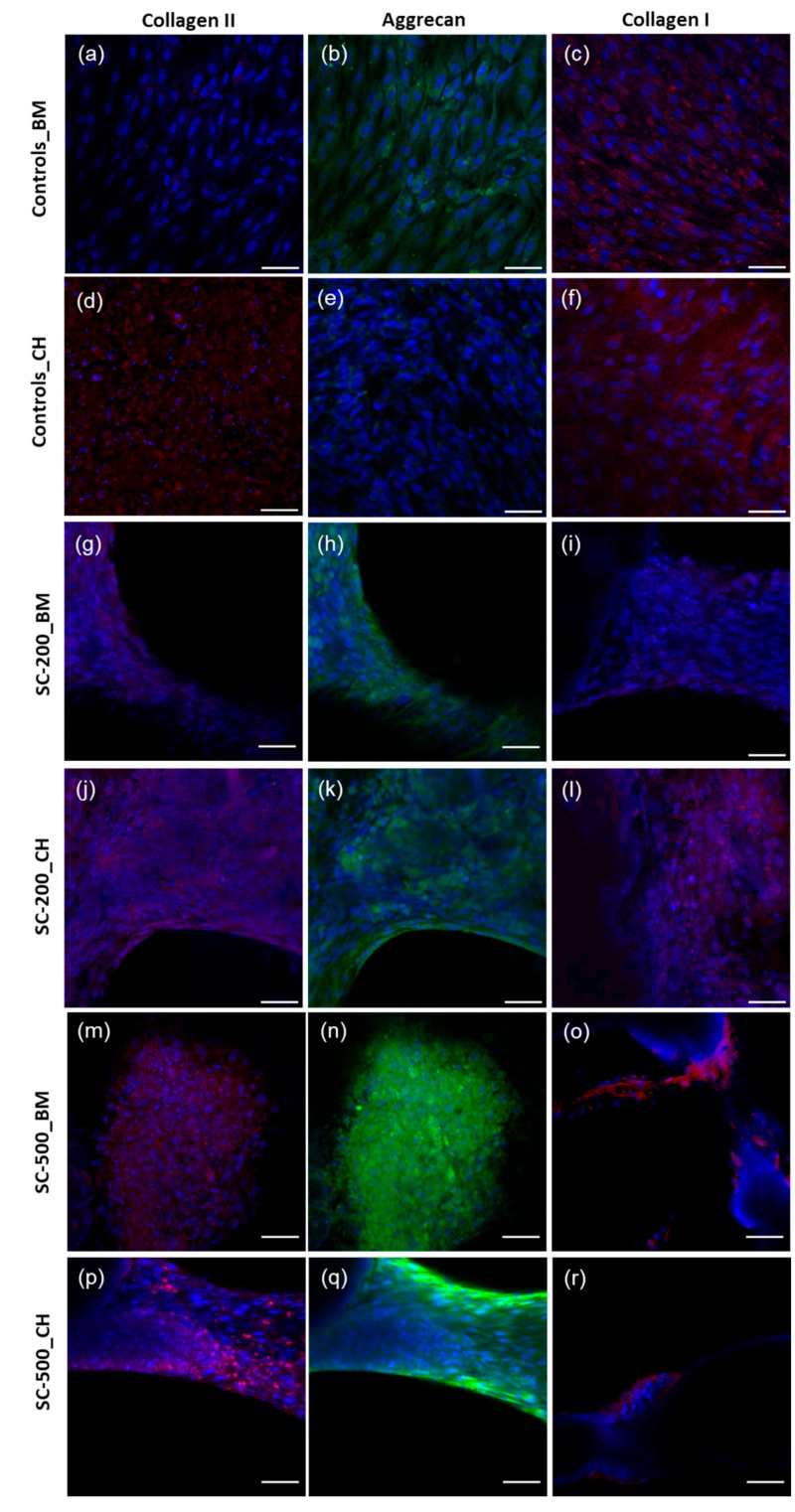
Representative immunohistochemical images of oBMSC on coverslips (**a**–**f**) and on SC-200 (**g**–**l**) and SC-500 (**m**–**r**) cultured for 21 days with chondrogenic (“_CH”) and basal (“_BM”) medium. COL2 is marked in red, ACAN in green, COL1 in red, and nuclei in blue. The scale bar is 66 µm.

**Figure 8:**
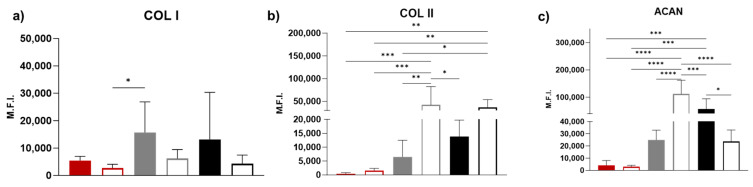
Quantitative analysis of mean fluorescence intensity (MFI) of (**a**) collagen type I, (**b**) collagen type II, and (**c**) aggrecan in 2D and on scaffolds (SC-200 and SC-500) on day 21. Data are reported as mean ± SD, *n* = 3. Comparison between groups was assessed by ordinary one-way ANOVA (*p* < 0.05) (* *p* < 0.05, ** *p* < 0.01, *** *p* < 0.001, **** *p* < 0.0001).

Ovine bone marrow stem cells spontaneously produced collagen I (Figure 7c) and aggrecan (Figure 7b) in tissue culture dishes, at day 21. In the presence of CH medium, collagen II expression visually increased (Figure 7d), but the difference was not significant (*p* > 0.05) (Figure 8b). The intensity of aggrecan staining in cells cultured on both SC-200 (Figure 7h) and SC-500 (Figure 7n) was the strongest; however, the presence of CH medium significantly increased the aggrecan production only on SC-200 scaffolds in comparison to the untreated controls (Figure 8c) (Figure 7b,e,h,k,n,q). Interestingly, SC-500 in BM had a significant higher production of aggrecan in comparison to the 2D in BM as well (Figure 8c).

The expression of COL2 increased when ovine stem cells, grown in 2D, were supplemented with a chondrogenic medium (Figure 8b) (Figure 7a,d,g,j,m,p). COL2 was found on both the SC-200 and SC-500 scaffolds, with an increased expression when chondrogenic medium was used (Figure 7j,p and Figure 8p).

COL1 expression appeared to be present on SC-200 and SC-500, but the difference was not significant compared to the 2D controls (*p* > 0.05) (Figure 7c,f,j,l,o,r and Figure 8a). Aggrecan and collagen I/II were observed in the cytoplasm and in the secreted matrix around the cells, with greater amounts seen in the extracellular matrix of cells grown on the scaffolds.

### 3.6. Sulfated Glycosaminoglycan Quantification

**Figure 9:**
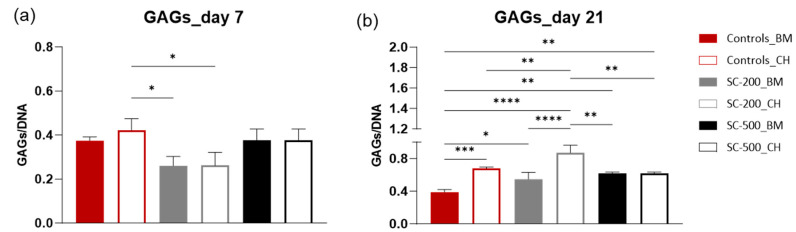
Analysis of sulfated GAGs content on day 7 (**a**) and day 21 (**b**) after oBMSC were seeded on scaffolds and coverslips. Total sulfated GAGs content was normalised against DNA content (n = 3). Comparison among groups was assessed by ordinary two-way ANOVA (* *p* < 0.05, ** *p* < 0.01, *** *p* < 0.001, **** *p* < 0.0001).

Normalised sulfated GAG content was measured on days 7 (Figure 9a) and 21 (Figure 9b). On day 7, a similar amount of GAGs was produced by oBMSC treated with different conditions. The only statistical differences were recorded for 2D controls in CH, whose GAG production was higher than SC-200 in BM and SC-200 in CH (*p* < 0.05). On day 21, instead, the overall amount of sulfated GAGs produced on SC-200 was significantly higher than the other groups (*p* < 0.05). For most of the samples, GAG content ratios increased with time: for 2D controls in CH (*p* < 0.001), for SC-200 in BM (*p* < 0.001), for SC-200 in CH (*p* < 0.0001), for SC-500 in BM (*p* < 0.001), and for SC-500 in CH (*p* < 0.001) (Figure S3). The results confirmed that the production of ECM markers, such as GAGs, increased on scaffolds, even without the use of exogenous growth factors.

## 4. Discussion

Ovine models are considered suitable for in vivo evaluation of osteochondral defects and scaffolds that are used to treat these defects, due to their physiological similarities to humans, including comparable body weight, bone formation, and anatomy. Furthermore, their ethical acceptance and ease of maintenance and handling enhance their suitability for preclinical studies [33]. For this reason, ovine models are often used in such investigations [42,43].

The in vitro evaluation of scaffolds used for microfracture tend to be carried out on hMSC [44,45,46]. It has been observed that human and ovine stem cells respond differently in terms of differentiation when exposed to growth factors [33,34,47]. Published studies are often contradictory, necessitating further research to decipher these preclinical models accurately. This study aims to validate the use of SiO_2_/PTHF/PCL-diCOOH scaffolds (with two different pore sizes) using ovine cells and to compare these results with the ones from our previous study on human stem cells and these constructs. This comparison seeks to ensure the scaffolds’ potential to facilitate the development of a cartilage tissue in sheep [32].

Comprehensive physical and mechanical characterisation of the SC-200 and SC-500 scaffolds has been previously published. Specifically, the authors showed that the hybrid materials present elastomeric deformation under tension and the ability to recover to the initial shape after the removal of the applied load. Furthermore, SC-200 presented a modulus of toughness of 142 kPa, whereas SC-500 presented a modulus of toughness of 60 kPa. Additionally, the modulus of elasticity of 200 µm pore size scaffolds was 7.2 MPa (in the for ε_c_ intervals of 5–10%) and 3.54 MPa for the 500 µm constructs. Overall, the studies indicate that the 200 µm porous materials are both stiffer and tougher than the 500 µm counterpart. Finally, for both the scaffolds, the struts diameter was between 140 to 200 µm [21,31].

Previous in vitro study on the differentiation of primary human bone marrow-derived mesenchymal stem cells (PCS-500-012^TM^, ATCC) on 3-D SiO_2_/PTHF/PCL-diCOOH scaffolds with specific channel sizes have been investigated. These analyses revealed that a channel width of 200–250 µm was optimal for promoting chondrogenic differentiation and maintaining the chondrocyte phenotype when cultured in chondrogenic media [32].

In this study, to enhance the cell adhesion to the scaffolds, we applied an FBS coating to the SiO_2/_PTHF/PCL-diCOOH scaffolds. Foetal calf serum, a component of standard cell culture medium, contains several cell adhesive proteins and has been successfully used to pre-coat surfaces (e.g., porous silicon, polystyrene, hydroxyapatite) to promote cell attachment, including the one of MSCs [48,49,50,51]. FBS supported oBMSC cell attachment by modifying the surface with integrin-binding domains, such as the tripeptide arginine–glycine–aspartate (contained, for instance, in fibronectin) [50,51]. Interestingly, in our study, pore size did not influence cell adhesion. Generally, when the pore areas are too large, there is a decrease in specific surface area, limiting cell attachment, and smaller porosities facilitate cell attachment [52]. However, in our study, the presence of FBS, combined with the seeding techniques and the initial high cell density, might have played an important role in ensuring an overall improved cell attachment.

The assessment of oBMSC proliferation revealed no difference between the two-pore sizes as well. Additionally, when compared to the 2D controls, live/dead staining showed that the scaffolds did not exhibit toxicity towards oBMSC. Proliferation remained constant across all groups over time. This phenomenon might be attributed to the cells reaching confluency by day 7. Notably, contact inhibition is known to reduce proliferation and growth [53] and plays a critical role in maintaining tissue homeostasis and preventing the overgrowth of cells. This inhibitory process is mediated by cell–cell adhesion molecules like Wnt signalling and Hippo, transmembrane proteins facilitating the adhesion of neighbouring cells. Upon reaching a certain cell density, these adhesion molecules become activated, thereby reducing proliferation and growth [54]. Furthermore, changes in metabolic activity might be due to cells undergoing different stages of differentiation, leading to diverse biosynthetic requirements and consequent fluctuations in metabolism [55,56].

SEM imaging conducted, on day 1, revealed a distinctive cellular morphology on uncoated scaffolds, characterised by a spherical phenotype. In contrast, cells on coated materials exhibited both rounded and elongated shapes. This might be driven by cell–scaffolds–protein interactions and material stiffness, as suggested in previous studies [31]. This morphology was maintained on all scaffolds treated with chondrogenic media on day 7. However, by day 21, cells on the SC-500 scaffold, even in presence of chondrogenic medium, reverted to a more fibroblastic morphology. This reversal may indicate a dedifferentiation of chondrogenic cells to elongated fibroblast phenotypes, as per previous reports [32,57,58]. These findings agree with what was previously reported by Li et al. and Nelson et al. for hMSC [21,59]. Instead, SC-200, supplemented with a chondrogenic medium, partially maintained their rounded morphology. Previous research has demonstrated that insufficient cell density can result in suboptimal synthesis of extracellular matrix (ECM) and the loss of the functional phenotype of chondrocytes [60,61]. In contrast, high-density cultures preserve the spherical shape of chondrocytes, although excessive density may impede nutrient exchange and reduce ECM production [60]. Nava et al. proposed that smaller pore dimensions may result in regions of localised hypoxia, which would favour chondrogenesis and hyaline cartilage production [62]. Therefore, it is possible that the pore size of the SC-500 scaffolds may be too large to support the critical cellular density, oxygen tension, and cell–cell/cell–scaffold–ECM interactions required for chondrogenic cell signalling pathways. Consequently, ovine bone marrow stem cells may have attached to the SC-500 and behaved more akin to those on plastic coverslips.

We then quantified the relative gene expression of early and late markers associated with chondrogenesis (*SOX5*, *SOX9*, *COL2A1*, and *ACAN*) and hypertrophy (*RUNX2* and *COL1A1*) [63]. *SOX9,* which binds to specific regions in the DNA, is the key regulator of chondrogenesis and is expressed in resting and proliferating chondrocytes, with maximum expression in pre-hypertrophic chondrocytes [64]. *SOX5* is a transcription factor expressed with *SOX9* during chondrogenic differentiation, activating *COL2A1* and *ACAN* genes in vitro [65]. While *SOX9* is necessary for chondrogenic differentiation, before and after mesenchymal condensations (initial stem cell aggregation into condensed clusters that is fundamental for cartilage formation), *SOX5* expression is seen at the start of stem cell differentiation into chondrocytes [63,64,65,66]. One of the key features of chondrogenesis is a temporal change in the composition of the deposited ECM [40]. During early condensation, matrix proteins such as fibronectin, collagen I, proteoglycan, and versican are prevalent. In contrast, the matrix deposited by differentiated chondrocytes is rich in collagen II and IX and the proteoglycan aggrecan [33,35,64]. Once the chondrocytes are differentiated, they can follow two pathways: one is to continue to proliferate and maintain their phenotype structure and function; the second is to undergo hypertrophy. Once the cells are hypertrophic, collagen X and collagen I are produced, and mineralisation of the matrix occurs [67]. Furthermore, at this stage, *RUNX2* is an early marker of osteogenesis, and MSC-derived osteochondroprogenitors express *RUNX2* during condensation of the skeletal anlagen [68]. Our results show that the SC-200 scaffold exhibited the highest expression of *COL2A1*, *SOX9, SOX5*, and *ACAN* when cultured in chondrogenic medium. On the other hand, the greatest expression of *RUNX2* on day 7 was observed in the SC-500 scaffolds in CH. However, the difference in expression between the scaffolds and 2D controls did not reach statistical significance. The highest expression of *RUNX2,* on day 21, was found in SC-200 in CH. Furthermore, we observed that the ratio of *SOX9* to *RUNX2* expression was highest in the SC-200 scaffolds under chondrogenic medium at both time points. *RUNX2* activity is usually repressed by *SOX9,* and it is known that if the *SOX9* expression is higher than *RUNX2*, MSC differentiate towards a chondrogenic phenotype [35]. Thus, our data suggest that SC-200 underwent a higher degree of chondrogenic differentiation, at gene level, than SC-500. Additionally, the ratio of *COL2A1* and *COL1A1* expression on day 7 was highest in SC-200 scaffolds cultured with chondrogenic medium. When comparing the fold-expression of chondrogenic markers between ovine bone marrow stem and human bone marrow stem cells on the same scaffolds, it was observed that ovine cells exhibited higher chondrogenesis at a genetic level [32]. For SC-500, *SOX9* had a similar fold change; *ACAN* and *COL2A1* were more expressed in oBMSC than hBMSC (ca. 47-fold versus ca. 7-fold change and ca. 5- versus 8-fold change, respectively); and *COL1A1* was similarly expressed. For SC-200, when comparing oBMSC with human bone marrow stem cells treated with chondrogenic medium, *SOX9* had a higher fold change in the ovine cells than their human counterpart (ca. 35 vs. 3); *ACAN* was more expressed, with ca. 234-fold change in oBMSC and ca. 3 in hBMSC; again, *COL2A1* was more expressed in oBMSC than in hBMSC (32 vs. 2), and *COL1A1* was similarly expressed. Overall, our results suggest that primary oBMSC are more predisposed to undergo chondrogenesis, at a genetic level, than hBMSC. Furthermore, this study shows, for the first time, that both SC-200 and SC-500 hybrid scaffolds can induce chondrogenic differentiation without the use of exogenous growth factors.

Immunohistochemical assessment revealed that primary ovine bone marrow stem cells, at passage 4, spontaneously synthesised collagen I and aggrecan when grown in basal cell culture medium. These findings are consistent with what was found by Mwale et al., who reported that human bone marrow stem cells may constitutively express aggrecan [68]. Furthermore, the expression of collagen I in bone marrow stem cells from varied species has been reported in several publications [69,70,71]. In our study, the addition of chondrogenic medium increased the expression of collagen II in oBMSC. SiO_2_/PTHF/PCL-diCOOH constructs, promoted a substantial deposition and accumulation of ECM components. Specifically, both collagen II and aggrecan (prevalent in articular cartilage) were found on the scaffolds, even in the absence of chondrogenic factors. Additionally, the negative marker for chondrogenesis, *COL1A1,* was present in the two channel widths of silica-PTHF/PCL scaffolds, especially when treated with CH. However, its expression seems to be pronounced in some areas of SC-500 cultured in chondrogenic medium. 

Normalised sulfated GAGs content measurements, on day 21, revealed a significantly higher concentration on SC-200 in CH compared to the other tested groups.

Overall, these findings suggest that SC-200 may promote a better proteoglycan production and chondrogenic markers expression, as previously seen in hBMSC [32]. Pore size plays a role in regulating chondrogenesis; smaller pores (200 µm) showed an overall better performance than larger pores (500 µm). This might have been possible because of several factors, such as cell clustering and aggregation, cell–cell interactions, and nutrient exchange. Smaller pores can create a hypoxic environment where chondrocytes strive, producing more ECM. Smaller pores can facilitate cell–cell interactions, too; clustering and aggregation in a 3D environment promote the development of cartilage tissue and allow for better paracrine signalling. Finally, different porosities can influence the mechanical properties of the scaffolds, influencing the maturation of cartilage tissue. However, in vitro studies on stem cells are not decisive in indicating the desirable pore sizes to promote chondrogenesis. For examples, pore sizes of 200 µm [71], 200–500 µm [69], and 860 µm [72] were reported to be desirable for chondrogenic differentiation of bone marrow stem cells.

While the precise mechanism leading to the induction of this chondrogenic differentiation remains to be determined, our results suggest that cell–cell and cell–scaffold (stiffness and surface chemistry) interactions might have played a role [25]. Future studies should also explore the potential contribution of silicon release from the hybrid scaffolds to the promotion of chondrogenesis of oBMSC. In an earlier study, scaffold biodegradation in PBS promoted a silicon release as low as 2.7 µg/mL after 7 days [35]. Previously, Ren et al. demonstrated that the addition of 200 ng/mL of sodium metasilicate (as a source of silica) to chondrogenic medium improved the expression of collagen II, aggrecan, the collagen type II/I ratio, the glycosaminoglycan contents, and the mechanical properties of mouse bone marrow scaffold-less 3D constructs [73]. However, while the osteogenic effects of silica have been extensively studied (increases osteoblast function, inhibits osteoclast function, promotes bone mineralisation, and induces vascular formation) [74], limited research has been conducted on the beneficial influence of silicon or silicate on chondrogenesis of stem cells.

This study has certain limitations, encompassing the examination of different cell densities, diverse coatings, resazurin assays at earlier time points, lower oxygen tensions, and more-specific markers of hypertrophy such as collagen, type X (ColX), matrix metalloproteinase (MMP13) and phosphorylated alkaline phosphatase (ALP) [75]. To enhance these results a higher number of cell donors are recommended due to the very high variability of cells isolated from individual sheep, as has previously been demonstrated [76]. Moreover, a more quantitative technique such as Western blotting should be utilised for measuring the quantities of collagens and aggrecan on the scaffolds. This choice is motivated by the potential impact of reflection and refraction of light on fluorescence microscopy, which could have caused artefacts in the images [77].

## 5. Conclusions

In conclusion, our study suggests that the SC-200 and SC-500 scaffolds can promote chondrogenesis in ovine bone marrow stem cells, even in the absence of chondrogenic growth factors. Furthermore, cells on SC-200 exhibited a higher expression of chondrogenic markers at a genetic level and better maintained a rounded phenotype (typical of chondrocytes) over the three weeks. These findings provide validation for further ovine studies. However, subtle differences in chondrogenesis exist between ovine and human MSC, with ovine cells tending to undergo chondrogenesis at a higher degree at the genetic level. It is noteworthy that this in vitro investigation could not fully emulate the physiological environment that cells experience in vivo, including mechanical loading or the presence of inflammatory factors. When considered in conjunction with previous studies, we recommend exploring the potential of the SiO2/PTHF/PCL-diCOOH hybrid scaffolds in ovine in vivo investigations to substantiate these findings. Furthermore, future studies should explore the influence of the released silicon on the induction of chondrogenesis of stem cells on SiO_2_/PTHF/PCL-diCOOH scaffolds.

## Data Availability

The raw data presented in this study are available on request from the corresponding author.

## Supplementary Materials

The following supporting information can be downloaded at https://www.mdpi.com/article/10.3390/bioengineering11020112/s1: Figure S1: SEM images representing oBMSC cultured on uncoated and FBS coated on day 1. Scale bar was 100 µm. Figure S2: Relative gene expression of (a) *RUNX2* and (b) *SOX9*; (c) *SOX9/RUNX2* ratio; relative gene expression of (d) *SOX5*, (e) *ACAN*, (f) *COL2A1* and (g) *COL1A1*; (h) *COL2A1/COL1A1* ratio. mRNA data are presented as fold change expression relative to the 2D controls grown with basal medium. Results are presented as a mean ± SD (n = 3). Comparison among groups was assessed by ordinary two-way ANOVA (*p* < 0.05) (* *p* < 0.05, ** *p* < 0.01, *** *p* < 0.001 **** *p* < 0.0001). Figure S3: Analysis of sulfated GAG content at day-7 and day-21 after oBMSC were seeded in scaffolds. Total sulfated GAGs content was normalised against DNA content (n = 3). Two-way ANOVA was carried out, p < 0.05. Comparison among groups was assessed by ordinary two-way ANOVA (* *p* < 0.05, ** *p* < 0.01, *** *p* < 0.001 **** *p* < 0.0001).
