# Supplementary figures and images for: Ovine Mesenchymal Stem Cell Chondrogenesis on a Novel 3D-Printed Hybrid Scaffold In Vitro
Source: Bioengineering (Basel). 2024 Jan 24;11(2):112. doi: 10.3390/bioengineering11020112 (PMC10886199; doi:10.3390/bioengineering11020112)

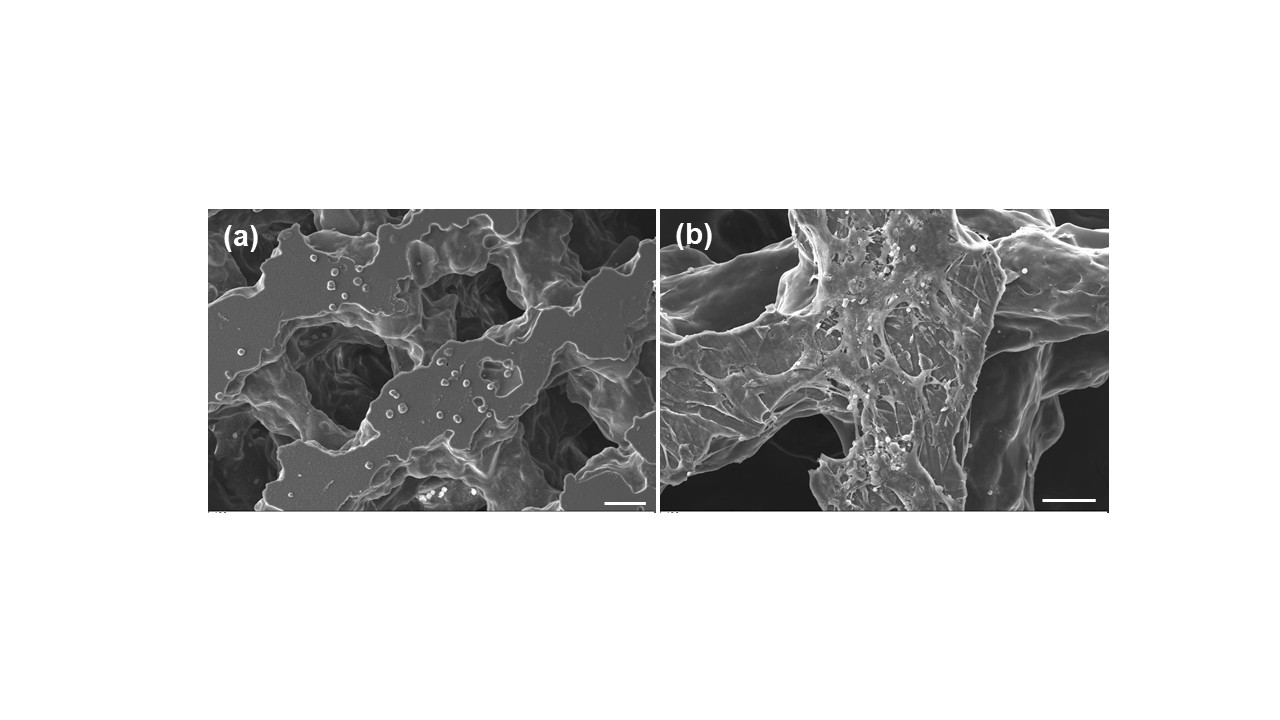

Supplement: Supplementary file 1 [file bioengineering-11-00112-s001.zip › Figure S1.tif]

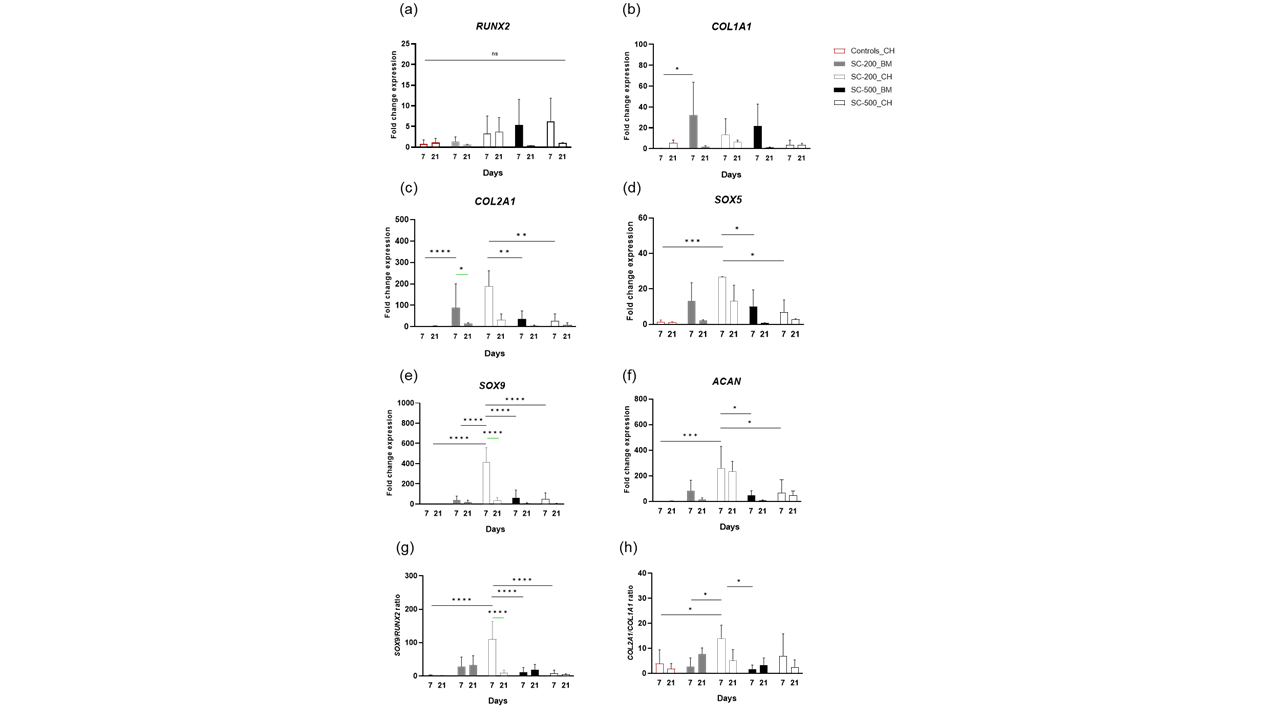

Supplement: Supplementary file 1 [file bioengineering-11-00112-s001.zip › Figure S2.tif]

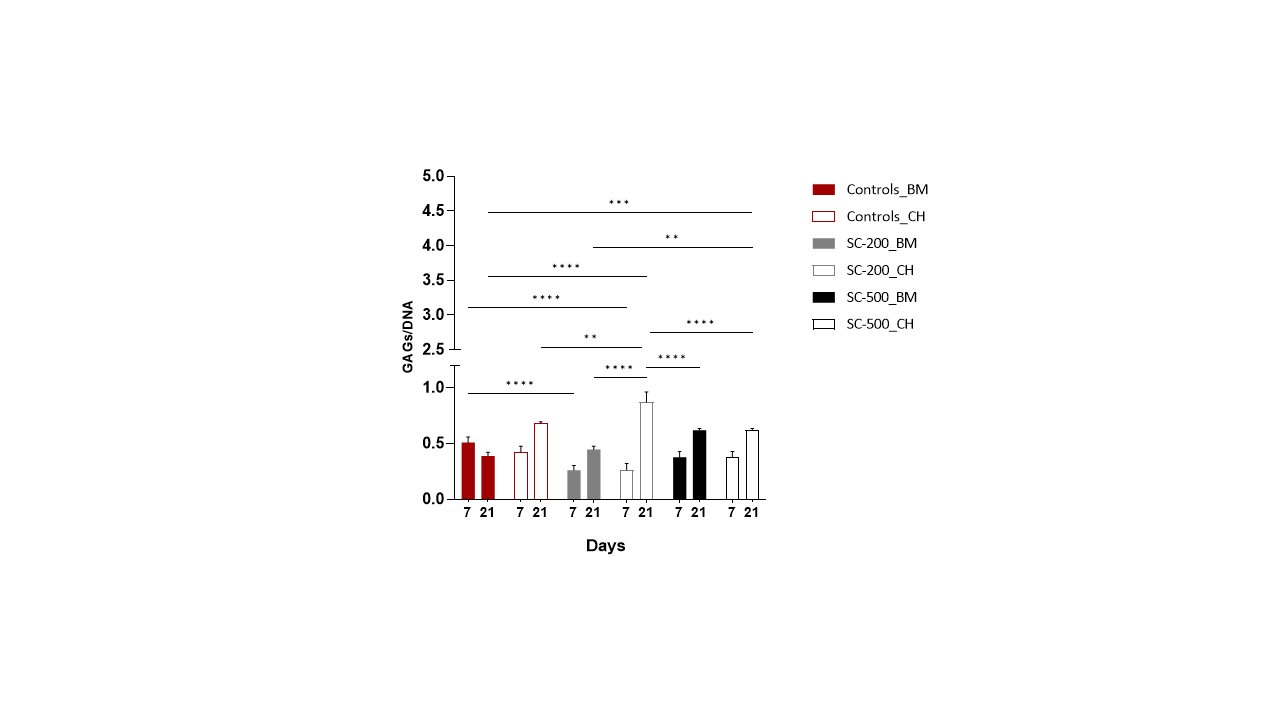

Supplement: Supplementary file 1 [file bioengineering-11-00112-s001.zip › Figure S3.tif]
